# Newborn screening of duchenne muscular dystrophy specifically targeting deletions amenable to exon-skipping therapy

**DOI:** 10.1038/s41598-021-82725-z

**Published:** 2021-02-04

**Authors:** Pablo Beckers, Jean-Hubert Caberg, Vinciane Dideberg, Tamara Dangouloff, Johan T. den Dunnen, Vincent Bours, Laurent Servais, François Boemer

**Affiliations:** 1 Biochemical Genetics Laboratory, Human Genetic Department, CHU de Liège, Université de Liège, CHU Sart-Tilman, Domaine Universitaire du Sart-Tilman, Avenue de l’Hôpital, 1, 4000 Liège, Belgium; 2 Molecular Genetics Laboratory, Human Genetic Department, CHU Sart-Tilman, University of Liege, Liège, Belgium; 3 Division of Child Neurology, Neuromuscular Reference Center Disease, Department of Pediatrics, University Hospital Liège & University of Liège, Liège, Belgium; 4 Department of Human Genetics and Clinical Genetics, Leiden University Medical Center, Leiden, The Netherlands; 5 Head of Human Genetics Department, CHU Sart-Tilman, University of Liege, Liège, Belgium; 6 Department of Paediatrics, MDUK Neuromuscular Center, University of Oxford, Oxford, UK

**Keywords:** Genetics, Molecular biology, Diseases, Molecular medicine

## Abstract

Duchenne Muscular Dystrophy (DMD) is a lethal progressive muscle-wasting disease. New treatment strategies relying on *DMD* gene exon-skipping therapy have recently been approved and about 30% of patients could be amenable to exon 51, 53 or 45 skipping. We evaluated the spectrum of deletions reported in DMD registries, and designed a method to screen newborns and identify *DMD* deletions amenable to exon 51, 53 and 45 skipping. We developed a multiplex qPCR assay identifying hemi(homo)-zygotic deletions of the flanking exons of these therapeutic targets in *DMD* exons (i.e. exons 44, 46, 50, 52 and 54). We conducted an evaluation of our new method in 51 male patients with a DMD phenotype, 50 female carriers of a *DMD* deletion and 19 controls. Studies were performed on dried blood spots with patient’s consent. We analyzed qPCR amplification curves of controls, carriers, and DMD patients to discern the presence or the absence of the target exons. Analysis of the exons flanking the exon-skipping targets permitted the identification of patients that could benefit from exon-skipping. All samples were correctly genotyped, with either presence or absence of amplification of the target exon. This proof-of-concept study demonstrates that this new assay is a highly sensitive method to identify DMD patients carrying deletions that are rescuable by exon-skipping treatment. The method is easily scalable to population-based screening. This targeted screening approach could address the new management paradigm in DMD, and could help to optimize the beneficial therapeutic effect of DMD therapies by permitting pre-symptomatic care.

## Introduction

Duchenne Muscular Dystrophy (DMD) is the most common and severe form of muscular dystrophy, marked by progressive muscle degeneration. DMD is caused by variants—mostly out-of-frame—in the *DMD* gene, which encodes for the protein dystrophin^1^. Dystrophin interacts with other proteins to maintain the integrity and structure of musculoskeletal fibers in skeletal and cardiac (i.e. striated) muscle. In DMD patients, pathogenic variants of the *DMD* gene lead to a complete lack of dystrophin production. Involvement of striated muscle begins in early childhood, generally before the age of three years and is predominantly observed in males. Becker Muscular Dystrophy (BMD) also involves the *DMD* gene, and is characterized by residual dystrophin production, so that BMD patients present later with a milder clinical phenotype^2^. Nearly all patients with BMD harbour an in-frame pathogenic variant/mutation.

Deletions of one or more exons of the *DMD* gene account for approximately 60–70% of pathogenic variants in patients with DMD and BMD^3^. Recently, novel therapeutic approaches to DMD involving exon skipping have been developed. These RNA-level therapies aim to skip the flanking exon of an out-of-frame mutation to transform it to an in-frame mutation, in order to induce the synthesis of a truncated and partially functional dystrophin protein^4,5^. Eteplirsen (Exondys) and golodirsen (Vyondys 53) were approved by the Food and Drug Administration (FDA) for the treatment of DMD in patients who carry a confirmed mutation that is amenable to exon 51 and exon 53 skipping, respectively^6,7^. Several other similar approaches, such as, suvodirsen, viltolarsen, casimersen, or rAAV-U7snRNA-E53^8^, targeting exons 51, 53 or 45, are or have been under evaluation to expand the spectrum of treatable DMD patients.

Apart from exon-skipping approaches, several gene therapy strategies are also under investigation. Ataluren (PTC124), an approved drug in Europe, enables ribosomal readthrough of premature nonsense mutations to produce full-length, functional dystrophin and has shown promising results in several studies^2,9^. Gene therapy using microdystrophin has proven efficacy in different canine models of DMD^10,11^. Three phase I/II clinical trials are also ongoing to assess the safety of AAV-microdystrophin intravenous injection (ClinicalTrials identifier: NCT03368742, NCT03769116, NCT03362502). Many other downstream therapeutic alternatives are currently under investigation, such as, upregulation of utrophin, using *GALGT2* gene therapy^12^, idebenone^13^, givinostat^14^, or edasalonexent^15^.

Considering the current advances in DMD treatment, newborn screening (NBS) programs for DMD are increasingly being considered^16–18^. Indeed, there is increasing awareness that DMD patients amenable to exon-skipping should be treated as early as possible (i.e. from birth) in order to maximize the beneficial therapeutic effect^16,17^. In keeping with this, a study of the safety and efficacy of eteplirsen is ongoing in children as young as six months (ClinicalTrials identifier: NCT03218995).

To date, technical aspects of population-based screening for DMD have been evaluated in pilot studies. These assessments systematically considered the quantification of creatine kinase (CK), or its muscular isoform (CKMM), on dried blood spots (DBS) as a primary marker of DMD^19–24^. CK is a marker of the disease process and does not directly reflect the genetic defect. Accordingly, both false negatives and false positives occur using either CK or CKMM assays as a first-line test. Another important consideration is that not all DMD patients would benefit from early treatment since current approved therapies only target specific defects of *DMD* gene. In addition, gene-transfer by adeno-associated virus that could cover all phenotype is not currently evaluated at birth, since the weight-limited dose that can be administered in a newborn would be severely diluted by growth, with no possibility to re-administer later in life.

Consequently, we have designed an assay for the early identification of DMD molecular defects that could be amenable to exon skipping therapies targeting exons 51, 53 and 45. This approach involves a multiplex quantitative polymerase chain reaction (qPCR) assay to identify hemi(homo)–zygotic deletions of exon 44, 46, 50, 52 and 54 of *DMD* gene (i.e. the flanking exons of exon-45, exon-51 and exon-53-skipping targets) on dried blood spots. We set out to identify 100% of treatable DMD patients that could benefit from early treatment. The method also leads to the identification of a small subgroup of DMD that cannot be treated by exon skipping; however, in those patients an early diagnosis is provided.

## Results

### Database review

**Figure 1:**
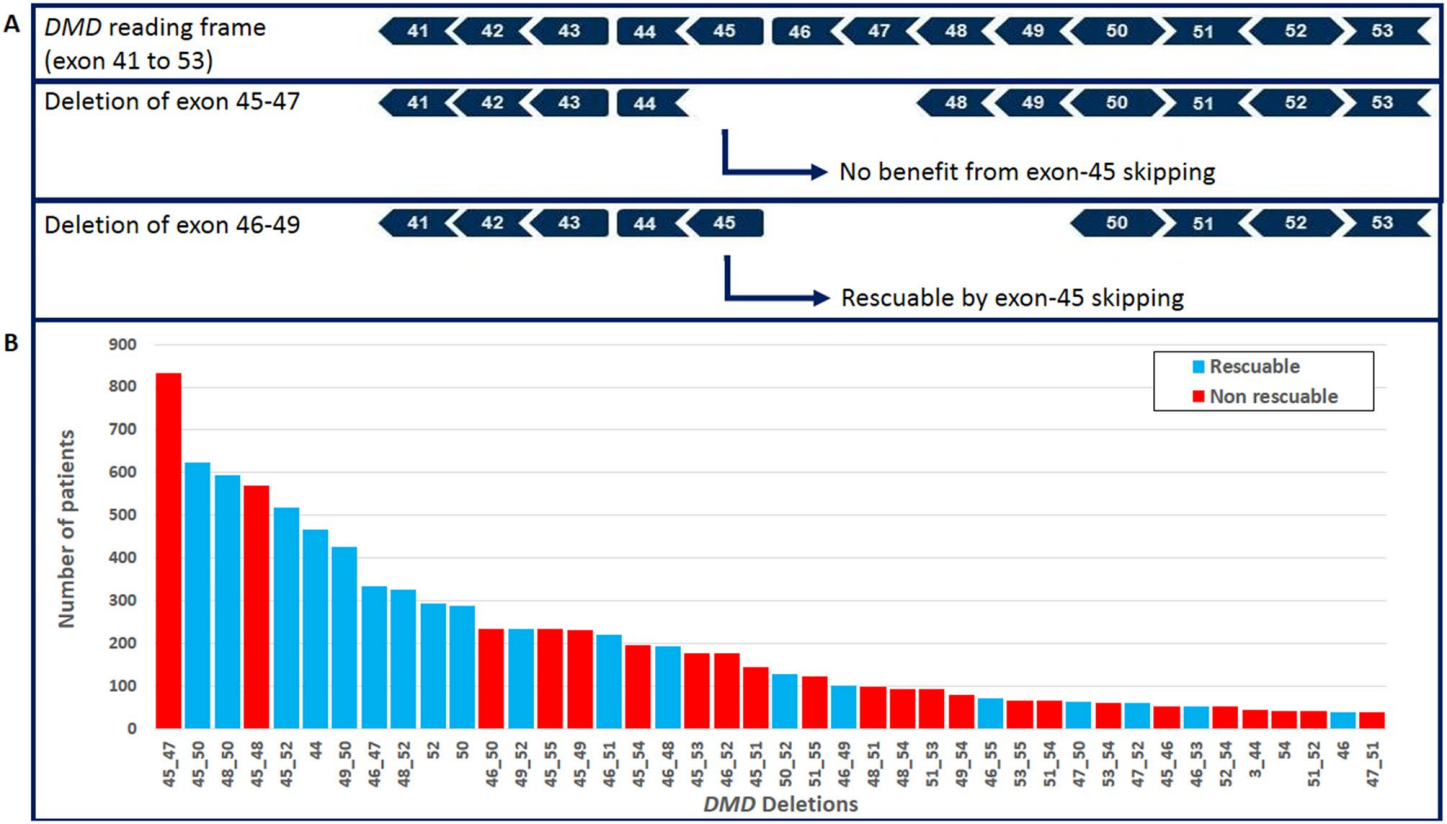
(**A**) Reading frame from exons 41 to 53 of *DMD* gene (upper frame). Example of the in-frame deletion of exons 45–47, which cannot benefit from exon-45 skipping (central frame). Example of the out-of-frame deletion of exons 46–49, which is amenable to exon-45 skipping (lower frame). (**B**) Number of patients with a large deletion (≥ 1 exon) covering at least one flanked exon reported in LOVD-DMD database. Deletions involving 35 or more patients are shown. Blue-bars point the deletions rescuable to either single exon-51, exon-53 or exon-45 skipping, red-bars correspond to deletion not rescuable by the aforementioned therapies.

Our approach was to identify patients amenable to exon-skipping therapy by screening for individuals with hemi(homo)-zygotic deletions of the flanking exons of exon-skipping targets. Identification of a deletion of any of these flanking exons does not, however, invariably involve a genotype that could benefit from these treatments. For example, a deletion of flanking exon 46 may indicate a deletion of exons 45–47, or a deletion of exons 46–49. The in-frame deletion of exons 45–47 would not benefit from exon-45 skipping, while the out-of-frame deletion of exons 46–49 can be rescued by skipping of exon-45 (Fig. 1A).

To determine the efficiency of our approach, we assessed the percentage of DMD patients reported in the LOVD-DMD registry that would be identified by our assay and would be rescuable by either exon-51, exon-53 or exon-45 skipping therapy (Fig. 1B). This analysis also shows that assaying the flanking exons is an effective approach to identify many patients that could benefit from these therapies (Table 1).

**Table 1:** Number of patients reported in LOVD-DMD database, and proportion of these patients that would be rescuable by either exon-51, exon-53 or exon-45 skipping.

	Number of patients	Number of patients that would benefit from either exon 45, 51 or 53 skipping (%)
Entries reported in database^a^	26,078	
Patients with a deletion of *DMD* gene	16,414	
Patients with a deletion covering at least one flanking exon	9532^b^	5144 (54.0)^c^
Patients with a deletion overlapping at least exon 44	1075	511 (47.5)
Patients with a deletion overlapping at least exon 46	5357	1016 (19.0)
Patients with a deletion overlapping at least exon 50	5494	2034 (37.0)
Patients with a deletion overlapping at least exon 52	3375	1583 (46.9)
Patients with a deletion overlapping at least exon 54	1386	0 (0.0)

^a^LOVD-DMD database accessed on April 20^th^ 2020.

^b^The numbers of patients with a deletion of at least one flanking exon corresponds to the number of DMD patients reported in LOVD-DMD database that would be identified by our assay.

^c^Of the 9532 DMD patients identified by our assay, 5144 would benefit from either exon 51, 53 or 45 skipping.

### qPCR results

We analyzed 120 samples with known genotypes. Deletion of any of the five target exons (exons 44, 46, 50, 52 and 54) of the *DMD* gene was characterized by the absence of amplification of the corresponding target. As shown in Table 2, the assay correctly identified all subjects, with either presence or absence of amplification of the target exon. To corroborate the absence of amplification, for each exon, an NFR below 0.2 was found as being discriminant between the deleted group and the carrier and control groups.

**Table 2:** Amplification results of the different subject groups: “deleted group”, “carrier group” and “control group”.

Subject group	Target *DMD* exon					Genotype^d^	Number of patients
	Exon 44	Exon 46	Exon 50	Exon 52	Exon 54		
Deleted group	Del*	Ampli*	Ampli	Ampli	Ampli	del_13-44	n = 34 (total)
Deleted group	Del	Ampli	Ampli	Ampli	Ampli	del_44	
Deleted group	Del	Ampli	Ampli	Ampli	Ampli	del_20-44	
Deleted group	Del	Ampli	Ampli	Ampli	Ampli	del_20-44	
Deleted group	Ampli	Del	Del	Del	Del	del_46-55	
Deleted group	Ampli	Del	Del	Del	Del	del_45-55	
Deleted group	Ampli	Del	Del	Del	Del	del_45-55	
Deleted group	Ampli	Del	Del	Del	Del	del_45-55	
Deleted group	Ampli	Del	Del	Del	Del	del_45-55	
Deleted group	Ampli	Del	Del	Del	Ampli	del_46-53	
Deleted group	Ampli	Del	Del	Del	Ampli	del 46–52	
Deleted group	Ampli	Del	Del	Ampli	Ampli	del_45-50	
Deleted group	Ampli	Del	Del	Ampli	Ampli	del_45-50	
Deleted group	Ampli	Del	Del	Ampli	Ampli	del_45-50	
Deleted group	Ampli	Del	Del	Ampli	Ampli	del_45-50	
Deleted group	Ampli	Del	Del	Ampli	Ampli	del_45-50	
Deleted group	Ampli	Del	Del	Ampli	Ampli	del_45-50	
Deleted group	Ampli	Del	Del	Ampli	Ampli	del_45-50	
Deleted group	Ampli	Del	Ampli	Ampli	Ampli	del_45-47	
Deleted group	Ampli	Del	Ampli	Ampli	Ampli	del_45-47	
Deleted group	Ampli	Del	Ampli	Ampli	Ampli	del_45-47	
Deleted group	Ampli	Del	Ampli	Ampli	Ampli	del_45-46	
Deleted group	Ampli	Del	Ampli	Ampli	Ampli	del_46-48	
Deleted group	Ampli	Del	Ampli	Ampli	Ampli	del_45-48	
Deleted group	Ampli	Del	Ampli	Ampli	Ampli	del_45-48	
Deleted group	Ampli	Ampli	Del	Del	Del	del_48-55	
Deleted group	Ampli	Ampli	Del	Del	Del	del_49-54	
Deleted group	Ampli	Ampli	Del	Del	Del	del_49-54	
Deleted group	Ampli	Ampli	Del	Ampli	Ampli	del_50	
Deleted group	Ampli	Ampli	Del	Ampli	Ampli	del_50	
Deleted group	Ampli	Ampli	Del	Ampli	Ampli	del_50	
Deleted group	Ampli	Ampli	Ampli	Del	Del	del_51-55	
Deleted group	Ampli	Ampli	Ampli	Del	Del	del_51-64	
Deleted group	Ampli	Ampli	Ampli	Del	Ampli	del_52	
Deleted group	Ampli	Ampli	Ampli	Ampli	Ampli	N/A^a^	n = 17
Carrier group	Ampli	Ampli	Ampli	Ampli	Ampli	N/A^b^	n = 32
Carrier group	Ampli	Ampli	Ampli	Ampli	Ampli	N/A^c^	n = 18
Control group	Ampli	Ampli	Ampli	Ampli	Ampli	Normal	n = 19

a = male patients with a *DMD* deletion not overlapping any of the 5 target exons.

b = female subjects carrying a *DMD* deletion overlapping at least one of the 5 target exons.

c = female subjects carrying a *DMD* deletion not overlapping any of the 5 target exons.

d = Deleted exons were identified through MLPA. Intronic breakpoints have not been sequenced; genotypes are reported according to the LOVD-DMD database.

*Results show if the target exon is deleted (Del) or amplified (Ampli).

*N/A* Not Applicable.

Within the “deleted group” (n = 51), there were 10 different deletion patterns seen: a deletion overlapping exon 44 (n = 4 patients), a deletion overlapping exon 46 to 54 (n = 5), a deletion overlapping exon 46 to 52 (n = 2), a deletion overlapping exon 46 to 50 (n = 7), a deletion overlapping exon 46 (n = 7), a deletion overlapping exon 50 to 54 (n = 3), a deletion overlapping exon 50 (n = 3), a deletion overlapping exon 52 to 54 (n = 2), a deletion overlapping exon 52 (n = 1), and patients with a deletion not overlapping any target exon (n = 17).

**Figure 2:**
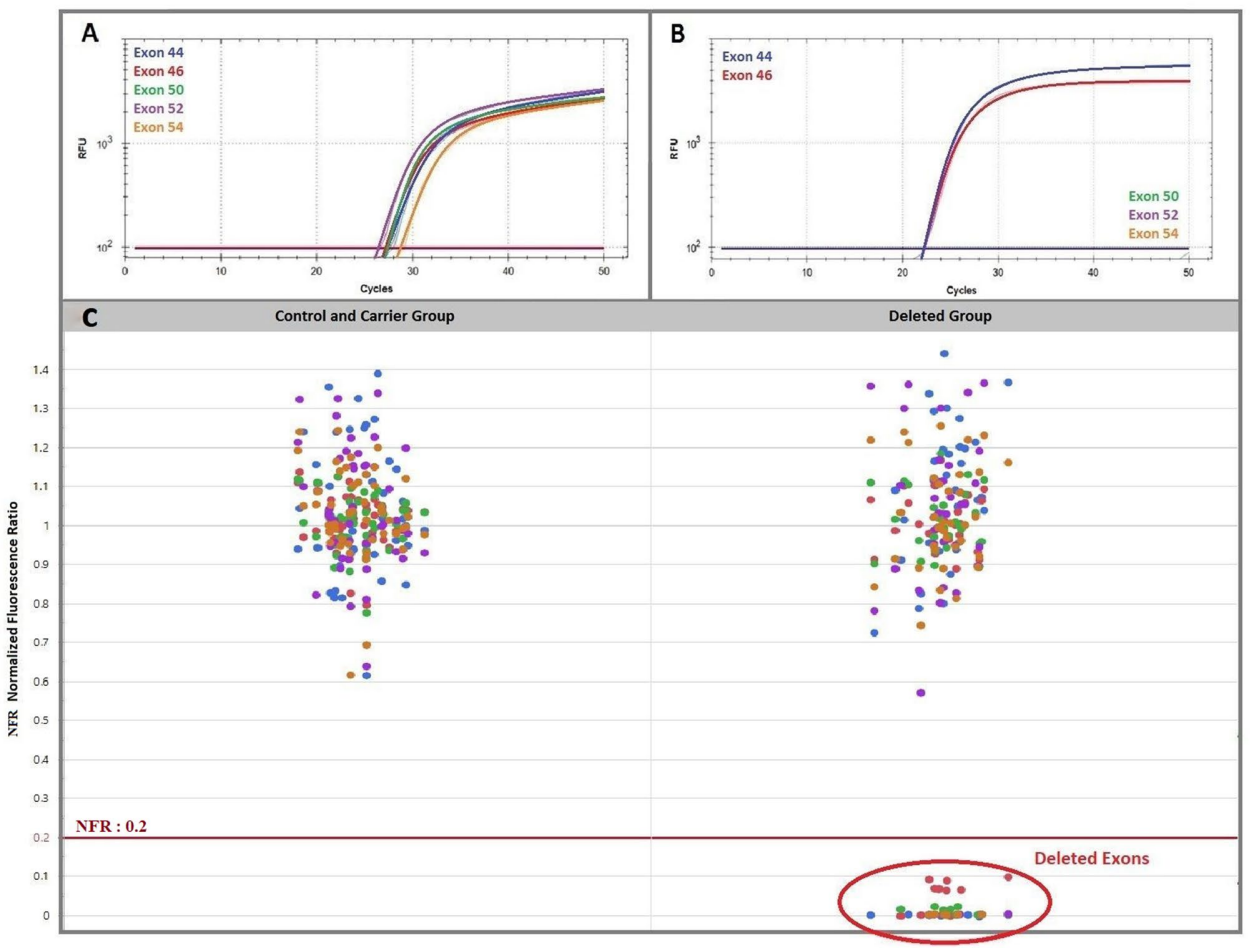
Amplification curves and scattered endpoint fluorescence of control group and carrier group, and DMD patients with a deletion of at least one target exon (deleted group). (**A**) Amplification curves of a patient without deletion of any target exons. (**B**) Amplification curves of a patient with a deletion of exons 50 to 54. (**C**) Normalized Fluorescence Ratio (NFR) of control and carrier group versus deleted group (i.e. DMD patients with a deletion overlapping at least one of the five target exons).

The 34 DMD patients of the “deleted group” with a deletion overlapping at least one of the five target exons were correctly characterized by an absence of fluorescence of the corresponding probes. The other 17 male patients of the “deleted group”, with a deletion of a *DMD* exon not targeted by our assay, presented a clear amplified profile of all target exons. All subjects of the female “carrier group” and the “control group” also were characterized by a normal significant fluorescent signal of each probe. Our assay did not discriminate between carrier females and controls. The amplification profile of each target exon is summarized in Fig. 2. The results demonstrate that the technique achieved 100% sensibility and 100% specificity in the population studied.

## Discussion

We show the feasibility and reliability of a simple test to detect newborns with DMD that would be eligible for exon skipping therapy. Our study is a proof-of-concept approach to NBS for DMD using a qPCR method. We set up a high-throughput assay to target deletions that are amenable to exon-51, exon-53 or exon-45 skipping treatments (i.e. screening for deletion of exons 44, 46, 50, 52 and 54 of the *DMD* gene). We analyzed 51 DMD patients, 50 female *DMD* gene deletion carriers and 19 healthy individuals without any deletion of the *DMD* gene. Each of the 120 subjects was correctly categorized by the assay with either the presence or absence of amplification of the target exon corresponding to their known genotype. Should a very rare variant affect the specificity of our primers and probes, we expect scoring a false positive result due to absence of amplification.

DMD is devastating muscle disease with an early onset. However, due to a 1.3–2.5 year delay in clinical diagnosis after the appearance of first symptoms, the mean age at diagnosis of DMD patients is 4.4 years^25–27^. Since early treatment is recommended^16–18^, reducing the delay before diagnosis might be aided via the inclusion of DMD in newborn screening performed under existing public health screening protocols. To the best of our knowledge, only exon-skipping is being studied to date in children as young as six months of age (ClinicalTrials identifier: NCT03218995). Pre-treating patients before gene therapy using an exon-skipping approach could potentiate the effect of gene therapy. Such pre-treatment would allow the use of lower-and therefore safer-doses of vector to bring about a higher level of dystrophin expression in the long term^28,29^.

The availability of personalized treatments represents a paradigm shift in the management of the subset of DMD patients that are amenable to their use, particularly as treatment can now be initiated pre-symptomatically. Deployment of our test raises some challenges as it addresses a subset of patients in which the use of an exon skipping therapy is possible. For those patients and their families, the benefit of early diagnosis of DMD is clear. For the large proportion of patients that are currently not suitable for these therapies, the ongoing development of gene therapy offers hope, albeit that significant clinical hurdles need to be overcome. For these patients, early diagnosis is still relevant to allow for the timely introduction of physiotherapy and corticosteroids. As part of evolving improvements in the treatment of DMD, we believe that our approach represents an effective and practical optimization of diagnosis. As therapeutic options expand, this test could also evolve as part of a more comprehensive DMD testing strategy when combined with other techniques.

Initiatives have previously been undertaken in various countries to explore the feasibility and efficacy of NBS for DMD^19–23^. Those studies, relying either on CK or CKMM quantitation, achieved modest sensitivity and specificity. The Wales DMD newborn screening program, for example, has carried out CK quantitation as a first-tier test on more than 340,000 neonates. The CK assay has shown a sensitivity of 81.6% with a positive predictive value of 38.6%^20^. More recently, a CKMM assay was evaluated in two independent populations in a limited number of newborns^23^. That study reported one false negative result on a premature infant. Authors also showed that both the gestational age and the age of the newborn at time of sampling affecting CKMM levels. This can become an issue when screening large populations with significant subgroups of preterm neonates, and could greatly impact the number of second-tier molecular tests required to confirm DMD diagnosis. Comparatively, while restricted to a small number of samples, our assay presented a sensitivity of 100%. Experience shows that qPCR assays dedicated to the NBS of spinal muscular atrophy (i.e. a technically similar assay) have very high sensitivities and specificities^30–32^. We suggest that there is a reasonable expectation that the DMD assay we describe could perform similarly in larger cohorts.

This study acts as a feasibility study for designing a subsequent wider NBS project for DMD. Technically, since our real-time PCR reader is equipped with five fluorescence channels, a two-step validation protocol is necessary. Results showing an amplification of either one or more exons are acceptable, as any amplified exon can act as a reference sequence. Samples showing no amplification for any of the five exons (i.e. any *DMD* deletion covering exons 44 to 54) would be re-run in a 2-plex qPCR reaction, combining each individual exon with the *RPP30* reference gene to confirm the deletion. In the context of neonatal screening, positive results would be confirmed on a fresh-EDTA sample using the MLPA assay, as the test is already commercially available. MLPA testing, combined with an analysis of the RNA transcript, is also mandatory to establish precisely the individual genotype, confirming whether a patient is eligible for exon-skipping therapy.

Regarding the experimental conditions, the qPCR assay we describe is highly practical, robust and easily scalable to population-based screening. It is also not economically burdensome (less than 5 euros per sample), considering that the DNA is already extracted for NBS of other molecular genetic diseases. These other targets include spinal muscular atrophy^33,34^ another devastating genetic condition without circulating diagnostic biomarkers for which innovative medications have dramatically changed the prognosis when administered early, and that has prompted several NBS programs^30–32^. In the future, societal and medical cost-effectiveness evaluations could be carried out on large population to compare the “qPCR/MLPA” protocol we describe with the two-tier “CKMM DMD genotyping” strategy proposed by Mendell et al.^24^. In the context of developing an NBS algorithm for DMD, our qPCR assay could also be considered as a second-line fast-track procedure to rapidly identify whether CKMM positive samples would be eligible for exon-skipping therapies.

Although DMD is expected to affect only male patients, and the costs of processing the sorting of samples by gender greatly exceed the low costs of analysis, we envisage performing screening on both male and female neonates. We could thereby identify the very rare cases of DMD in women with co-existing Turner syndrome^35^. On the other hand, a few cases of female carriers expressing a DMD phenotype have been described in situation of X-chromosome inactivation (XCI) skewing^36^. Our assay is not intended to identify carriers of a *DMD* deletion, and females with a DMD phenotype related to non-random patterns of XCI would be missed.

DMD patients eligible for either exon-51, exon-53 or exon-45 skipping treatments account for approximately 30% of DMD cases^37^. With an incidence evaluated at about of 1/5,000 males in the general population^38^, approximately 1/15,000 males could be identified using our approach, which is far above other commonly accepted disorders in NBS programs. Our multiplex PCR is not intended to detect all deletions in the *DMD* gene nor can it identify duplications or point mutations. A negative result on the current assay does therefore not definitively rule out a diagnosis of DMD, since about 70% of DMD patients are not eligible for either exon-51, exon-53, or exon-45 skipping therapies and would not be identified by our assay. Nevertheless, the spectrum of DMD genotypes potentially rescuable by single or double antisense-mediated exon skipping is constantly growing. The actual applicability of exon skipping approach, assessing which exon(s) should be skipped to restore the open reading frame in *DMD* gene, could theoretically benefit 83% of patients with DMD^37^. Should any future DMD therapies targeting other exons or specific variants be developed, our method could be easily scaled-up to target *DMD* exons other than 51, 53 and 45, thereby extending its applicability to a wider panel of *DMD* genotypes. qPCR technology has some limitations and cannot identify the entire panel of variants described in the *DMD* gene. Next-generation sequencing (NGS) represents the ultimate approach to screen newborns for DMD. Notwithstanding, NGS is currently too expensive to be used for full population screening for the foreseeable future.

Our methodology could raise some ethical concerns regarding DMD patients who would be left out by our assay. Our approach is, however, not the first initiative of NBS prompted by innovative therapies that misses some cases affected by a particular disease. Indeed, NBS for spinal muscular atrophy is nowadays largely accepted amongst pediatricians, families and advocacy groups, although screening assays specifically focus only on the identification of homozygotic deletions of the *SMN1* gene and miss *SMN1* heterozygotic and *SMN2* deletions. Screening for spinal muscular atrophy using this method was added to the Recommended Uniform Screening Panel (RUSP), the official list of disorders which US public health departments use to screen newborns, despite the availability of only one approved treatment. Type 0 spinal muscular atrophy patients were, therefore, identified by screening, but could not benefit from any specific treatment available at that time.

The DMD population diagnosed at birth with our test accounts for approximately 9532/26078 (36.5%) in the LOVD database. Overall, 5144/26078 (19.7%) could be offered gene therapy. The remaining 4388/26078 (16.8%) that are diagnosed with exon deletions but are not amenable to current therapy, also have significant potential benefits stemming from early diagnosis. In this latter group, physiotherapy and steroids could be implemented years earlier than the current situation in which they are simply waiting for DMD to manifest clinically. Currently, physiotherapy and corticosteroids are typically initiated about the age of 4 years, even in patients diagnosed earlier. At that time, more than half of patients will have been diagnosed^39^. NBS of all DMD patients could reduce diagnosis journey and anticipate familial recurrence, but since the age at diagnosis is not the main driving factor for initiating disease-specific treatment like steroids, there is no more rationale to screen today all DMD patients than any recessive rare disorders, which explain why DMD is not usually part of NBS programs. The approval of new disease-modifying treatment brings a new insight for a subset of patients with a deletion amenable to treatment. The introduction of our novel screening test would thus not negatively impact the current management of DMD cases, while adding an easy, non-expensive, and efficient way to detect 100% of the patients that are eligible for gene therapy.

One of the limitations of this approach could be the development of non-mutation specific treatments such as microdystrophin. Nevertheless, gene therapy as a one-shot injection will probably not be applicable before the age of two years, and is currently in trials after the age of 4. The risk of a dilution effect through growth could limit the treatment of newborns. In this context, the pre-treatment of eligible exon skipping therapy patients from birth until microdystrophin injection could help to potentiate gene therapy treatment^40^.

## Conclusion

In conclusion, we provide proof-of-concept of a test that can potentially detect DMD patients at birth that are eligible for currently approved exon skipping 51, 53 and 45 therapies. The development of this assay was driven by the approval of exon-skipping drugs in DMD management. Our test addresses a significant paradigm shift in NBS, by identifying newborns amenable to a specific available treatment, rather than detecting all newborns with a particular disease. This method meets a specific current -and possibly transitory- need in DMD care, focused on the identification of patients that could benefit from exon-skipping therapies. Early identification of DMD holds promise for preserving functional performance in affected children via the initiation of treatment at a young age. Should the current assay’s performance be further validated in larger studies, it could act as a means to improve DMD patient identification and management in the clinical setting for countries where exon-skipping drugs are available.

## Materials and methods

### DMD registry analysis

We reviewed the Leiden Open Variation Database for the *DMD* gene (LOVD-DMD), that currently contains more than 26,000 patient entries^41^. We evaluated the spectrum of reported deletions to provide a theoretical rationale for the development of an assay identifying deletions that could benefit from either exon-51, exon-53 or exon-45 skipping therapies.

### Subjects

Blood from DMD and control samples were collected as dried blood spots (DBS). *DMD* gene profile of all patients was performed using Multiplex Ligation-Dependent Probe Amplification (MLPA) assay in the course of diagnostic workup.

In total, 120 samples were collected and were classified into three different subgroups:

The “deleted group” consisted of 51 male patients with a clinical DMD phenotype. Amongst them, 34 had a confirmed deletion of one or more *DMD* exons amenable to exon-skipping targeted therapies (i.e. exons 44, 46, 50, 52 or 54). The other 17 male patients carried a deletion of another *DMD* exon.

The “carrier group” included 50 females with one deleted *DMD* allele, of whom 32 carried a deletion that overlapped at least one of the target exons 44, 46, 50, 52 or 54, while 18 had a deletion of any other *DMD* exon.

The “control group” consisted of 19 normal individuals with no DMD deletion/duplication or point mutations.

### qPCR Technical design

The DMD genotyping assay was designed and validated to detect hemi-(homo)-zygotic deletions of *DMD* exons 44, 46, 50, 52 and 54. The protocol uses a multiplex quantitative polymerase chain reaction (qPCR) assay.

DNA was extracted from one 3.1-mm dried blood spot according to the protocol described previously in^33^. Isolated DNA was not quantitated, and 1 µL of freshly extracted DNA was mixed with 5 × Takyon master mix (Eurogentec), primers and probes in a total volume of 25 µL. The sequences and concentrations of primers and probes are shown in Table 3. Primers and probes have been designed to have the fewest possible described polymorphisms (Supplemental Table 1).

**Table 3:** qPCR primers and probes used to amplify and identify *DMD* Exons 44, 46, 50, 52 and 54.

Target	Sequence	Concentration (nmol/L)
***DMD exon 44***		
Forward primer	5′- TAC CTG CAG GCG ATT TGA C -3'	300
Reverse primer	5′- CAC CCT TCA GAA CCT GAT CTT T -3'	300
Probe	5′- FAM—AAA TTC CTG AGA ATT GGG AAC ATG—BHQ -3'	130
***DMD exon 46***		
Forward primer	5′- TTT ATG GTT GGA GGA AGC AGA -3'	300
Reverse primer	5′- AAT GGG CAG AAA ACC AAT GA -3'	300
Probe	5′- YakimaYellow—AAC CTG GAA AAG AGC AGC AAC T—BHQ -3'	90
***DMD exon 50***		
Forward primer	5′- CTG AGT GGA AGG CGG TAA AC -3'	300
Reverse primer	5′- TCT CAC CCA GTC ATC ACT TCA -3'	300
Probe	5′- ROX—ACTTCAAGAGCTGAGGGCAAAG—BHQ -3'	80
***DMD exon 52***		
Forward primer	5′- AAT ACA CAA CGC TGA AGA ACC C -3'	300
Reverse primer	5′- TTG TGT GTC CCA TGC TTG TT -3'	300
Probe	5′- Atto 647 N—CGC TGC CCA AAA TTT GAA AAA—BHQ -3'	150
***DMD exon 54***		
Forward primer	5′- TCT ATA GCA GTT GGC CAA AGA C -3'	300
Reverse primer	5′- TCA TGG TCC ATC CAG TTT CA -3'	300
Probe	5′- Atto 700—AAT ATC AAT GCC TCT TGG AGA AGC—BHQ -3'	180

Assays were run on a CFX96 Real-Time PCR System (Biorad) under the following conditions: 95 °C for 5 min, followed by 40 cycles of 95 °C for 30 s and 61 °C for 75 s.

### Interpretation of findings

To normalize amplification results and to facilitate the interpretation, a normalized fluorescence ratio (NFR) was calculated dividing the endpoint fluorescence of each sample by the median endpoint fluorescence of the corresponding exon of all samples of the same run.

### Ethics

The study was approved by the local ethics committees and patients or guardians provided written informed consent (2019/278 and CEH 84/19). All experiments were performed in accordance with relevant guidelines and regulations.

### Ethics approval and consent to participate

The study was approved by the local ethics committees and patients or guardians provided written informed consent (2019/278 and CEH 84/19). All experiments were performed in accordance with relevant guidelines and regulations. Hôpital Universitaire des Enfants Reine Fabiola (ULB): Members: Mr F Devaux, Dr J Groswasser, Mrs G Hendrijckx, Mrs K Van Aerschot, Mrs N Andersson, Mr P Lemaire, Dr H Demanet, Dr A Ferster, Dr C Fonteyne, Dr C De Laet, Dr D Biarent, Dr V Vlieghe, Dr P Simoni, Mrs L Lambotte. CHU Liege: Members: Dr V Seutin, Dr J Demonty, Dr G Daenen, R Agirman, Dr E Baudoux, Dr A Blavier, Dr F Caeymax, Mme MN Englebert, Dr P Firket, Mme I Hemans, Dr M Lamy, Dr M Lejeune, Mr P Lissens, Mme P Modanese, Dr AS Parent, Dr M Radermecker, Dr R Radermecker, Mme I Roland, Dr I Ruten, Mme C Thirion.

## Supplementary Information

Supplementary Information

## Data Availability

The datasets used and/or analyzed during the current study are available from the corresponding author on reasonable request.

## Abbreviations

DMD: Duchenne muscular dystrophy
BMD: Becker muscular dystrophy
qPCR: Quantitative polymerase chain reaction
MLPA: Multiplex ligation-dependent probe amplification
