# Supplementary material for: Newborn screening of duchenne muscular dystrophy specifically targeting deletions amenable to exon-skipping therapy
Source: Sci Rep. 2021 Feb 4;11:3011. doi: 10.1038/s41598-021-82725-z (PMC7862591; doi:10.1038/s41598-021-82725-z)
Supplement: Supplementary file 1 — Supplementary Information [file 41598_2021_82725_MOESM1_ESM.docx]

**Newborn Screening of Duchenne Muscular Dystrophy Specifically Targeting Deletions Amenable to Exon-skipping Therapy**

Pablo BECKERS, Jean-Hubert CABERG, Vinciane DIDEBERG, Tamara DANGOULOFF, Johan T. den DUNNEN, Vincent BOURS, Laurent SERVAIS, François BOEMER

**Supplemental Table.**

Minor allele frequency reported in gnomAD^*^ for polymorphisms identified in primers and probes sequences

| **Target** | | **Variant ID** | **Minor Allele Frequency** |
| --- | --- | --- | --- |
| Exon 44 | Forward primer | rs774305637 | 5.52E-06 |
|  |  | rs128626250 | Mutation (stop gained) |
|  | Reverse primer | rs1447528109 | 4.57E-05 |
|  | Probe | rs886043157 | Mutation (frameshift) |
|  |  | rs765726373 | 5.51E-06 |
|  |  | rs761965562 | 5.51E-06 |
|  |  | rs1023328955 | 2.46E-05 |
|  |  | rs753999355 | 1.05E-04 |
| Exon 46 | Forward primer | rs753677881 | 5.46E-06 |
|  |  | rs757026131 | 1.09E-05 |
|  |  | rs797044743 | Mutation (frameshift) |
|  | Reverse primer | / | / |
|  | Probe | rs1274712958 | 5.46E-06 |
|  |  | rs142531761 | 1.37E-04 |
|  |  | rs145993057 | 5.46E-06 |
| Exon 50 | Forward primer | rs149003002 | 5.45E-06 |
|  |  | rs771992420 | 2.18E-05 |
|  | Reverse primer | rs183429765 | 2.27E-04 |
|  | Probe | rs797044764 | Mutation (frameshift) |
| Exon 52 | Forward primer | rs761741300 | 5.45E-06 |
|  |  | rs1429687609 | 5.46E-06 |
|  |  | rs765117527 | 1.09E-05 |
|  |  | rs750239623 | 9.75E-06 |
|  |  | rs373462513 | 7.96E-06 |
|  | Reverse primer | rs767215025 | 5.45E-06 |
|  |  | rs1161049283 | 4.57E-05 |
|  |  | rs752779998 | 6.34E-05 |
|  |  | rs1312650242 | 5.45E-06 |
|  |  | rs876657452 | 7.96E-06 |
|  |  | rs760058465 | 1.64E-05 |
|  | Probe | rs1466680131 | 5.45E-06 |
|  |  | rs748309027 | 1.09E-05 |
|  |  | rs369583884 | 2.73E-05 |
|  |  | rs778412539 | 1.95E-04 |
| Exon 54 | Forward primer | rs1388762712 | 5.46E-06 |
|  |  | rs746092688 | 1.09E-05 |
|  |  | rs772132098 | 1.46E-05 |
|  |  | rs775555418 | 3.28E-05 |
|  |  | rs1437840142 | 4.56E-05 |
|  |  | rs760693222 | 4.57E-05 |
|  |  | rs1279450648 | 4.56E-05 |
|  |  | rs886044016 | 5.46E-06 |
|  | Reverse primer | / | / |
|  | Probe | rs771081514 | 1.09E-05 |
|  |  | rs139254609 | 5.46E-06 |
|  |  | rs1395638836 | 9.75E-06 |
|  |  | rs371437381 | 5.46E-06 |
|  |  | rs767634310 | 1.09E-05 |

^*^ Genome aggregation database v2.1.1 (gnomad.broadinstitute.org), accessed on April 21^st^ 2020
